# Endoscopic ultrasound-guided selective inflow vessel devascularization and multi-point venous occlusion for gastric varices due to left-sided portal hypertension

**DOI:** 10.1055/a-2971-5252

**Published:** 2026-10-15

**Authors:** Jingtong Huang, Xiaohui Ruan, Side Liu, Kangmin Zhuang

**Affiliations:** 1 Guangdong Provincial Key Laboratory of Gastroenterology, Institute of Gastroenterology of Guangdong ProvinceDepartment of Gastroenterology 198153 Nanfang Hospital, Southern Medical UniversityGuangzhouGuangdongChina

Left-sided portal hypertension is a rare extrahepatic portal hypertension triggered primarily by splenic vein stenosis or obstruction, rather than liver cirrhosis. It characteristically causes numerous, large-caliber gastric varices with high bleeding risk. 1​ For patients ineligible for surgery, minimally invasive endoscopic therapy remains the mainstay for acute bleeding. However, it carries a high recurrence rate. 2​ ​3​ In this case, we performed endoscopic ultrasound–guided selective inflow vessel devascularization (EUS-SIVD) and multi-point venous occlusion to achieve precise hemostasis ( Video 1 ).

**Video 1** Endoscopic ultrasound–guided selective inflow vessel devascularization and multi-point venous occlusion for gastric varices from left-sided portal hypertension.


A 50-year-old male patient developed recurrent upper gastrointestinal bleeding from splenic vein compression caused by peripancreatic effusion following severe pancreatitis ( Fig. 1a, b ). Endoscopic ultrasound (EUS) revealed extensive large-caliber variceal masses in the gastric fundus and body.

**Fig. 1:**
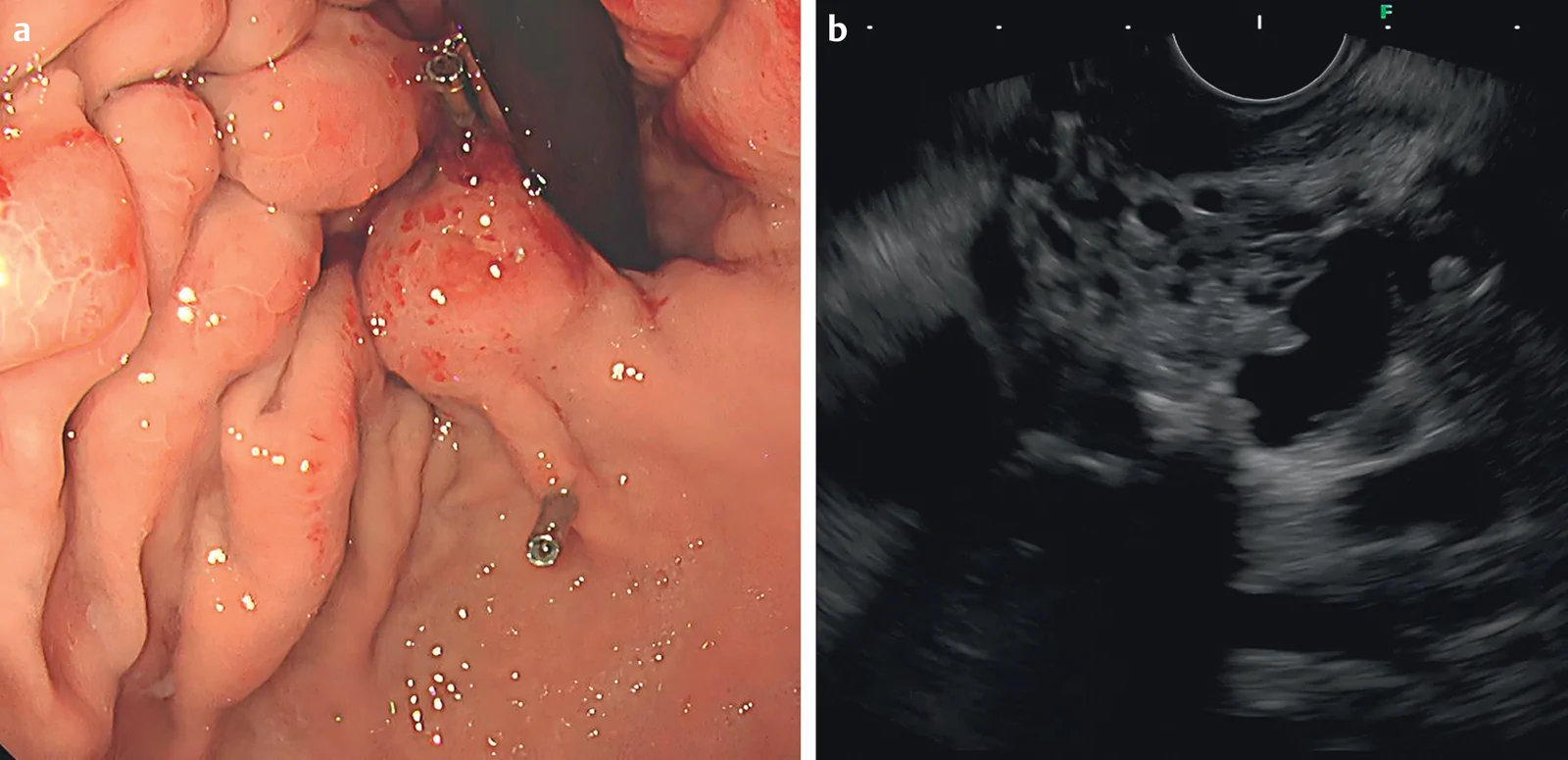
Images before treatment. ( **a** and **b** ) Before treatment, the patient had large varices in the gastric fundus and body, and many honeycomb-like vascular shadows could be seen under endoscopic ultrasound.

Under EUS guidance, we performed EUS-SIVD with multi-point obliteration of the afferent venous branches. A COOK 19G puncture needle was used to access three afferent venous branches, with one coil deployed at each site (two MWCE 35-14-20 coils and one MWCE 35-14-14 coil; Cook Medical, Bloomington, Indiana, USA). For the thicker variceal collateral branches, a “hypertonic glucose–cyanoacrylate–hypertonic glucose” sandwich method was adopted. After each injection of cyanoacrylate, 50% of hypertonic glucose was injected to flush the tract. Because of the large submucosal venous caliber and four prior failed endoscopic treatments, the same injection strategy was reinforced with coil embolization (MWCE 35-14-20, Cook Medical, Bloomington, Indiana, USA) for two superficial veins, so as to achieve a more secure therapeutic effect. All injections were performed through the same needle. Post-procedure color Doppler confirmed complete flow interruption with no active bleeding ( Fig. 2a, b ). One-year follow-up revealed a favorable prognosis ( Fig. 3 ), with hemoglobin levels steadily increasing ( Fig. 4 ) and no rebleeding or complications.

**Fig. 2:**
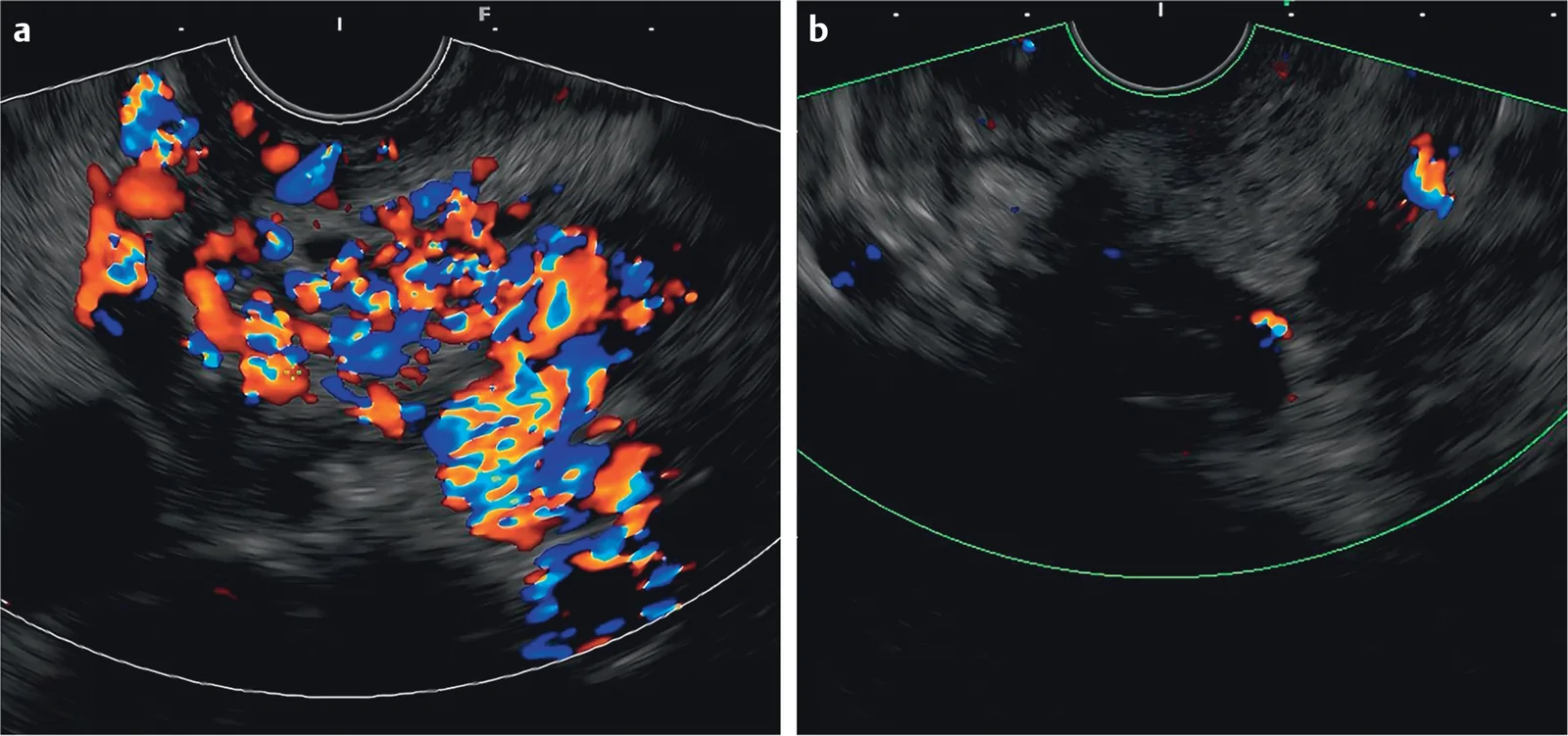
Endosonographic images. ( **a** ) Before treatment, multiple large, tortuous varices can be seen in the fundus and body of the stomach, and color Doppler imaging shows abundant blood flow signals. ( **b** ) After the treatment procedure, the blood flow signal is basically blocked.

**Fig. 3:**
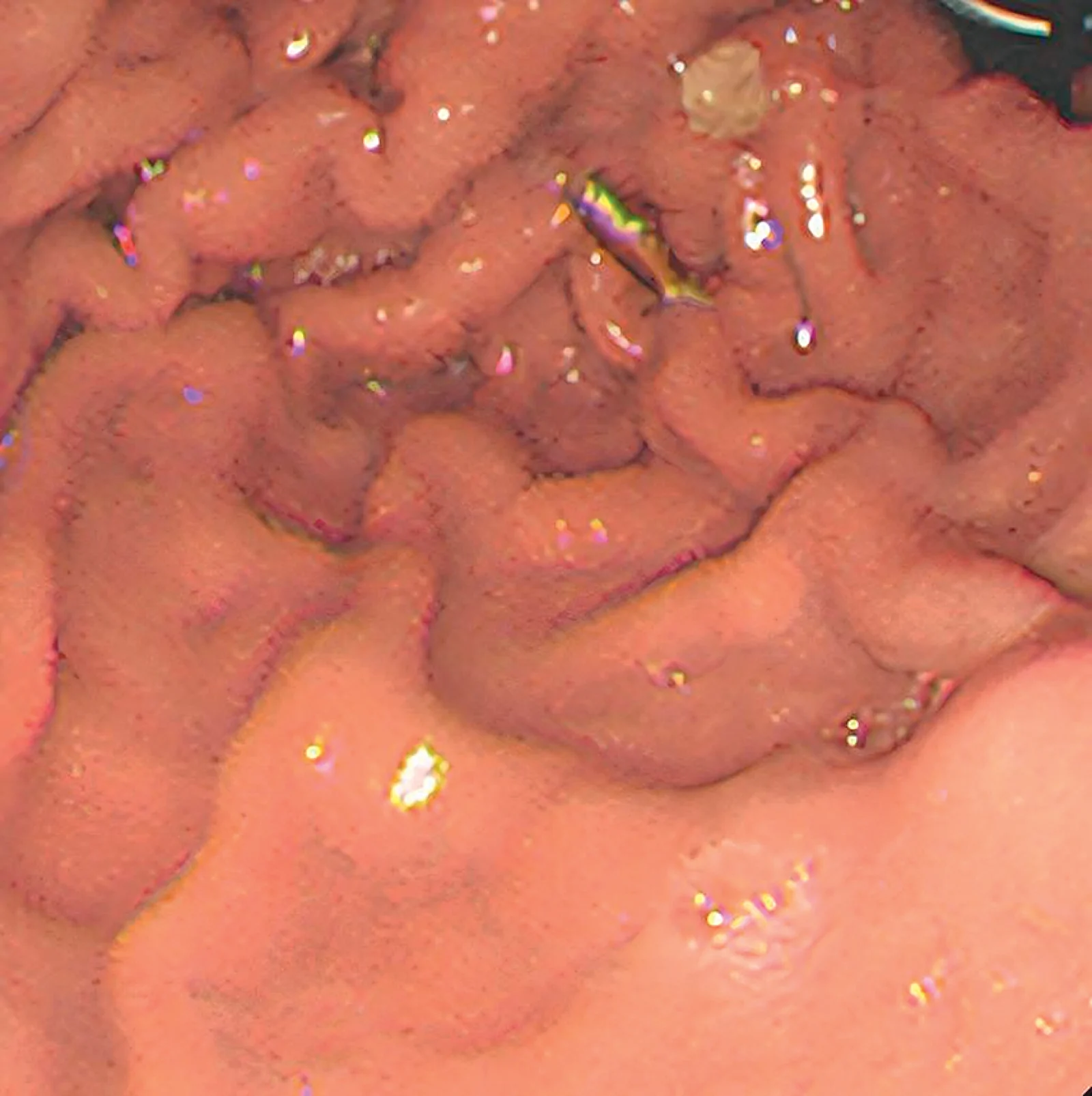
The follow-up imaging 1 year later showed a significant reduction in varicose veins.

**Fig. 4:**
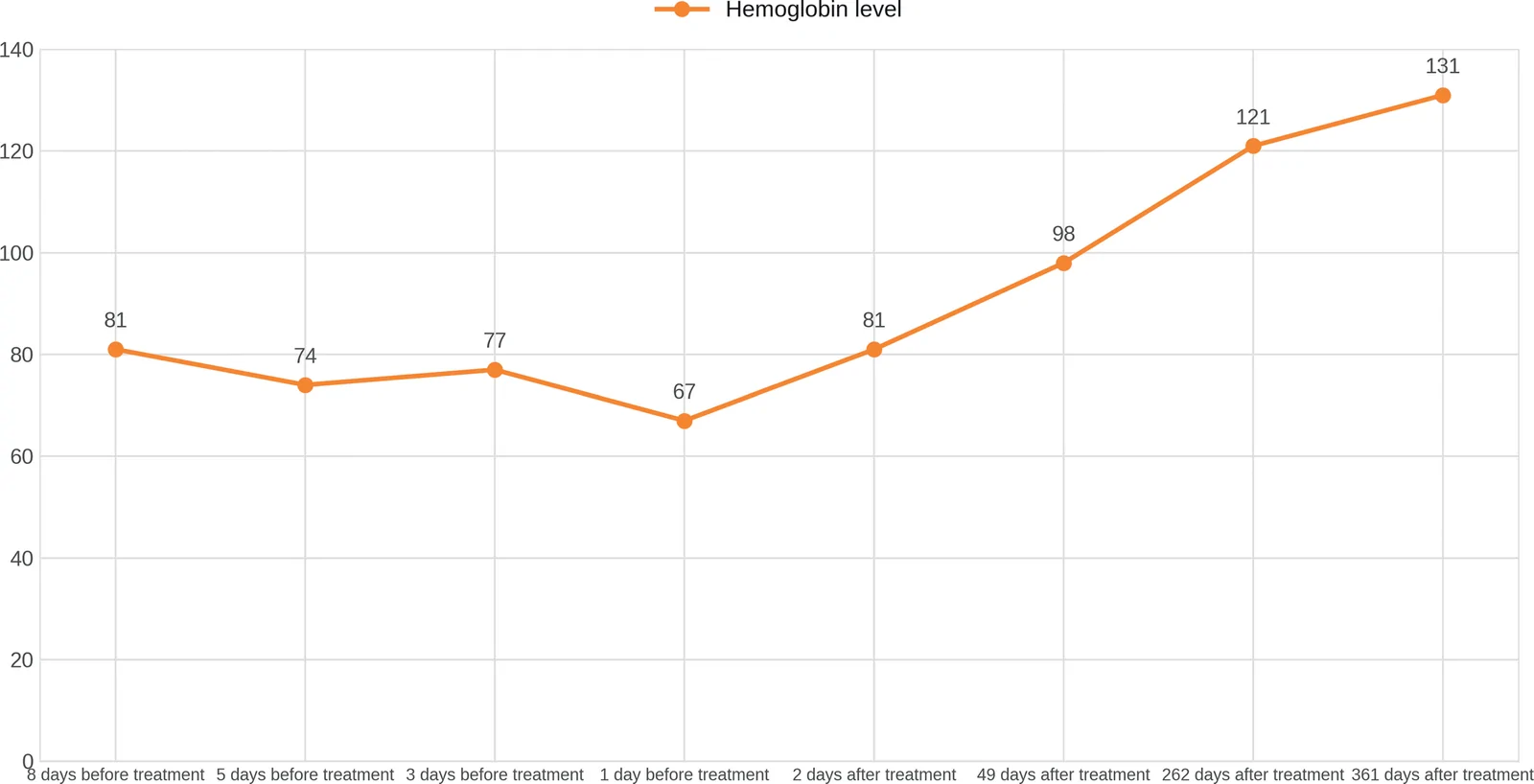
The trend of the patient's hemoglobin level changes within 1 year of follow-up.

EUS-SIVD with multi-point venous occlusion thoroughly hemostatizes extensive gastric varices while reducing cyanoacrylate dose, lowering post-procedural abdominal pain and glue-related ulcer risks with superior safety and low recurrence.

Endoscopy_UCTN_Code_TTT_1AS_2AL

## Declaration of GenAI Use

During the preparation of this manuscript, we use WorkBuddy for language polishing, and no generative artificial intelligence participated in data processing or other research work. We take full responsibility for the integrity and accuracy of the publication.
